# Breaking barriers in cancer pain management: a report from the National Week Against Cancer Pain in Argentina 2025

**DOI:** 10.3332/ecancer.2026.2153

**Published:** 2026-06-23

**Authors:** Ariel Cherro, Laura Aresca, María Susana Ciruzzi, Alejo Agranatti, María Fernanda Montaña, Cynthia Frahne, Jaqueline Cimerman

**Affiliations:** Palliative Care Council, Argentine Society of Medicine (SAM) , Gascón 655 11º “E” (CP 1181) Ciudad Autónoma de Buenos Aires (CABA), Argentina

**Keywords:** cancer pain, palliative care, national awareness week, pain management, volunteer support

## Abstract

**Introduction:**

Cancer-related pain remains a major public health challenge in Latin America, where systemic, professional and social barriers frequently delay access to adequate palliative care.

**Methods:**

The Palliative Care Council of the Argentine Society of Medicine organised the National Week Against Cancer Pain 2025, the second edition of a nationwide initiative first launched in 2022. The programme combined digital advocacy, community-based educational activities and a volunteer‑led clinical network. Mass-media communication was amplified by public figures, alongside in‑person and virtual activities for the community and free clinical consultations delivered through a dedicated web platform supported by institutional digital channels.

**Results:**

During the peak activation period, the campaign achieved nearly 50,000 direct digital impressions, with substantial additional reach generated through secondary dissemination by high‑profile influencers. More than 100 free clinical consultations were provided, both virtually and in person, across 13 Argentine provinces. Community‑based activities were conducted in multiple regions, supported by academic societies, provincial governments and civil organisations. The initiative generated over 50 appearances in national and regional media outlets.

**Conclusion:**

A coordinated strategy combining digital innovation, community engagement and professional volunteerism can significantly reduce barriers to cancer pain management. This experience offers a scalable and replicable model for improving early access to palliative care in middle‑income countries.

## Introduction

Pain is the most prevalent and feared symptom among people living with cancer. Despite the availability of clinical guidelines and essential medications, many patients in Argentina and across Latin America continue to experience avoidable suffering due to fragmented healthcare systems, delayed referrals to specialist services, insufficient professional training in pain and opioid management and persistent social misconceptions regarding analgesic use [1, 2]. In this context, ‘microsocial barriers’ refer to beliefs and attitudes within families and communities that normalise suffering, associate opioid use with addiction or imminent death or discourage open discussion about pain and symptom control.

Following its first edition in 2022, the *National Week Against Cancer Pain* was held for the second time in 2025, consolidating a growing national public health initiative aimed at addressing these barriers through accessible, interdisciplinary and community-oriented strategies.

The authors recognise that palliative care is a comprehensive, person-centred approach that extends beyond pain management and oncology and that its principles should be applied across a wide range of chronic and life-limiting non-oncological conditions [3]. However, this campaign deliberately focused on cancer-related pain as one of the most distressing and highly prevalent symptoms, using it as an entry point to address an urgent unmet need while simultaneously increasing the visibility and understanding of palliative care among the public, healthcare professionals and institutions.

## An integrated model: digital advocacy and community engagement

A central pillar of the initiative was its open and participatory communication model. Collaboration with nationally recognised artists, musicians, journalists and digital influencers enabled the translation of complex clinical concepts into a widely accessible message: the relief of cancer-related pain is a fundamental human right.

Digital engagement was generated through a dedicated website (www.sindolorporcancer.com.ar), supported by coordinated dissemination across institutional Instagram and Facebook platforms.

### Impact evaluation and metrics

Digital impressions and engagement metrics were collected using native analytics from institutional social media platforms (Instagram and Facebook) and website traffic monitoring tools. Metrics included number of impressions, reach, shares, comments and link clicks during the peak activation period. Media appearances were documented through systematic monitoring of national and regional press coverage. While these channels demonstrated strong direct interaction, the overall reach of the campaign was substantially expanded through secondary sharing via influencers’ personal accounts, generating hundreds of thousands of additional views beyond primary analytics.

### Community-based activities

Beyond digital communication, the initiative prioritised in-person and virtual activities for the community, adapting interventions to local contexts across the country. Activities included:

- Community talks and an artistic encounter in Benito Juárez, integrating health education with cultural expression.
- Training sessions for volunteers and medical students at the Italian University Institute of Rosario, followed by the installation of an outreach information tent along the Rosario waterfront, where trained teams engaged directly with the public.
- Meetings with adult and paediatric oncology non-governmental organisations in Mar del Plata (two separate activities).
- Community and institutional meetings in Chubut, San Rafael (Mendoza), Chaco and Jujuy, adapted to regional needs.
- A joint meeting with young oncologists in Córdoba, organised in collaboration with the Association of Clinical Oncologists of Córdoba (AOCC).

These activities reinforced the federal scope of the initiative and facilitated meaningful dialogue between healthcare professionals, patients, families and communities in diverse sociocultural settings.

## Volunteer clinical network and consultations

The initiative also delivered direct clinical impact. A dedicated digital platform functioned as a triage and coordination hub, connecting individuals requesting assistance with a federal network of volunteer physicians specialising in oncology and palliative care. All consultation requests were registered via a structured online form. Once the initial contact was established, clinical encounters were conducted independently by volunteer physicians according to their own records. To preserve patient-physician confidentiality, the organisation did not require reporting on specific clinical outcomes; thus, assistance followed its standard course as an autonomous medical relationship.

Over the course of the campaign, more than 100 free clinical consultations were provided through virtual and in‑person modalities, spanning 13 Argentine provinces. The most frequent issues addressed included uncontrolled cancer pain, without specific identification of the pain mechanism, doubts about access and coverage of palliative care and also cases of clinical improvement with palliative care and expressions of gratitude. When necessary, patients were referred to local palliative care services, oncology teams or pain specialists within their province. In regions lacking specialised services, cases were referred for telemedicine assistance with specialists from other regions and recommendations focused on optimising primary care-based symptom management and facilitating communication with treating physicians to ensure continuity of care. This model enabled rapid access to expert guidance and symptom management, effectively circumventing administrative, geographic and socioeconomic barriers commonly faced by patients.

## Institutional collaboration and academic endorsement

The project was developed within the Palliative Care Council of the Argentine Society of Medicine, in close collaboration with multiple scientific and professional organisations. Academic endorsement was provided by:

- *e*cancer
- Argentine Association of Clinical Oncology (AAOC)
- Argentine Association for the Study of Pain (AAED)
- Association of Clinical Oncologists of Córdoba (AOCC)
- Argentine Association of Geriatrics and Gerontology (SAGG)
- Argentine Association of Palliative Medicine and Palliative Care (AAMyCP)
- Instituto Pallium Latinoamérica
- Fundación Clínica 25 de Mayo
- Clínica 25 de Mayo
- Pain Care Center
- Association of Internal Medicine of Rosario (AMIR)

The initiative also received support from several provincial governments, strengthening its institutional legitimacy and territorial implementation.

### Media impact

The campaign achieved broad media visibility, with more than 50 appearances in national and regional media outlets. Appearances included national television interviews, regional radio programmes and digital health journalism platforms with combined audiences estimated in the hundreds of thousands. This exposure contributed to increased public awareness and helped reframe cancer pain as a preventable source of suffering rather than an inevitable consequence of disease.

## Discussion

The *National Week Against Cancer Pain 2025* illustrates how coordinated strategies – combining digital communication, community engagement, clinical volunteerism and institutional collaboration – can produce meaningful clinical and social impact. Similar public awareness initiatives linking media advocacy with direct clinical access have been described in high-income countries [5–7]; however, few reports from middle-income settings document integration of awareness campaigns with volunteer-based clinical networks at a national scale [1, 2, 5].

From a scalability perspective, the model required three key resources: (1) a coordinated volunteer professional network, (2) a centralised digital communication platform and (3) institutional endorsement to facilitate territorial implementation. Potential barriers to replication include limited availability of trained palliative care professionals, unequal digital access in rural areas and persistent regulatory or cultural barriers related to opioid prescribing. Practical lessons learned include the importance of early media engagement, collaboration with local civil organisations and the need for simple triage mechanisms to rapidly connect patients with appropriate expertise.

This initiative was primarily designed as an access and awareness strategy rather than a formal clinical research project. Although basic consultation data were collected, systematic follow-up, referral outcome tracking and patient-reported outcome measures were not prospectively implemented. Future editions of the campaign may incorporate structured evaluation frameworks to assess longitudinal clinical impact.

Importantly, the initiative demonstrates that awareness campaigns can be directly linked to concrete care delivery, fostering earlier integration of palliative care.

Persistent limitations in opioid availability, delayed integration of palliative care and inequitable access to specialist services continue to represent major challenges across Latin America, reinforcing the need for innovative, community‑oriented and scalable models such as the one described here [3, 4].

## Conclusion

This Argentine experience highlights the value of interdisciplinary and participatory public health strategies in cancer pain management. By aligning medical societies, healthcare professionals, community organisations, institutions and media voices, it is possible to expand access to pain relief, humanise oncological care and reduce avoidable suffering in middle‑income settings.

## Conflicts of interest

The authors declare no conflicts of interest.

## Funding

The initiative received limited, non‑conditional financial support from Carehome to cover operational communication costs (website development and community management). The sponsor had no role in the design, content, clinical activities or outcomes of the project.

## Use of artificial intelligence

Artificial intelligence-based tools were used solely to assist with language editing and stylistic refinement of the manuscript. These tools did not generate, modify or influence the scientific content, data interpretation or conclusions. All content and interpretations remain the sole responsibility of the authors.

## References

[1] Cleary J, Powell RA, Munene G (2013). Improving pain relief in developing countries: a cost and palliative care inventory. Ann Oncol.

[2] Hui D, Bruera E, De Lima L (2018). Barriers to integration of palliative care in oncology: perspectives from Latin America. Lancet Oncol.

[3] Pastrana T, De Lima L, Wenk R (2020). Atlas of Palliative Care in Latin America 2nd edn (IAHPC).

[4] Wenk R, Bertolino M, De Lima L (2021). Opioid availability and access to cancer pain management in Argentina. J Clin Oncol.

[5] World Health Organization (2021). Assessing the Development of Palliative Care Worldwide: a Set of Actionable Indicators.

[6] Centeno C, Sitte T, De Lima L (2007). The palliative care challenge: analysis of barriers and opportunities to integrate palliative care in the health system. J Palliative Med.

[7] Sallnow L, Richardson H, Murray SA (2016). The impact of a new public health approach to end-of-life care: a systematic review. Palliative Med.

